# Subjective Well-Being Among Unaccompanied Refugee Youth: Longitudinal Associations With Discrimination and Ethnic Identity Crisis

**DOI:** 10.3389/fpsyg.2022.920657

**Published:** 2022-08-25

**Authors:** Brit Oppedal, Serap Keles, Espen Røysamb

**Affiliations:** ^1^Division of Mental and Physical Health, Department of Child Health and Development, Norwegian Institute of Public Health (NIPH), Oslo, Norway; ^2^Knowledge Centre for Education, University of Stavanger, Stavanger, Norway; ^3^PROMENTA Research Center, Department of Psychology, University of Oslo, Oslo, Norway

**Keywords:** subjective well-being, unaccompanied refugee minors, youth, discrimination, ethnic identity, longitudinal, life satisfaction

## Abstract

Unaccompanied refugee youth (URY), who as children fled their countries to seek asylum in a foreign country without the company of an adult legal caretaker are described as being in a vulnerable situation. Many of them struggle with mental reactions to traumatic events experienced pre-migration, and to the daily hassles they face after being granted asylum and residence. Despite continuous high levels of mental health problems URY demonstrate remarkable agency and social mobility in the years after being granted asylum in their destination countries. A sense of subjective well-being (SWB) may enable resilient outcomes in people exposed to past or ongoing adversities. To fill the gap in the research literature about positive psychological outcomes among URY, the overall aim of this study was to explore the longitudinal associations between SWB and two taxing acculturation hassles: perceived discrimination and ethnic identity crisis. Three annual waves of self-report questionnaire data were collected from a population-based sample of URY; *n* = 581, *M*_*age*_ = 20.01(*SD* = 2.40), *M*_*length of stay*_ = 4.63 (*SD* = 4.40), 82 % male, mainly from Afghanistan, Somalia, Iraq, and Sri Lanka. The longitudinal associations between SWB, perceived discrimination and ethnic identity crisis across time were analyzed using auto-regressive cross-lagged modeling. The results revealed that perceived discrimination, but not ethnic identity crisis, negatively predicted subsequent levels of SWB. More importantly, high levels of SWB at one timepoint predicted decreases in both discrimination and ethnic identity crisis at subsequent timepoints. Further, increases in SWB from one timepoint to the next was associated with significant co-occurring decreases in both discrimination and ethnic identity crisis, and vice versa. Despite the negative effect of perceived discrimination on SWB, promoting SWB in URY can protect them from future hazards of acculturation hassles in complex ways. We underscore the need for more research on SWB among URY and other refugee youth. We further discuss the potential of SWB to foster resilient outcomes in young refugees and suggest that interventions to strengthen SWB among them should consider their transnational and multicultural realities and experiences.

## Introduction

Unaccompanied refugee youth (URY) are former unaccompanied asylum-seeking and refugee minors who have been granted residence and have resettled in their destination countries without the support of family caretakers, some of them reaching the age of maturity in the process. They are in an extremely vulnerable situation as a consequence of the accumulated pre-migration traumatic events they have been exposed to (Bean et al., 2007; Jensen et al., 2013; Vervliet et al., 2014), the high level of post-migration daily hassles they experience, and the lack of intimate caretakers that can support their adaptation to a new unfamiliar culture on a daily basis (Seglem et al., 2014; Keles et al., 2018). Despite experiencing high and stable levels of mental health problems (Vervliet et al., 2014; El Baba and Colucci, 2018; Keles et al., 2018), these are ambitious youth with strong agency and ability to succeed in their countries of resettlement (Oppedal et al., 2017; Horgan and Ní Raghallaigh, 2019). There is substantial over time social mobility among them: 79% of all URY between 18 – 29 years of age were either in employment or education four years after they have received residence, compared to 85% of the overall population in the same age-groups (Dalgard et al., 2018). Considering that URY are in a vulnerable situation, and the time it takes to learn a new culture and language, these findings about agency, ambitions, participation, and social mobility are intriguing. They call for more research that may elucidate their positive functioning despite the high level of adversities they are exposed to.

There is considerable knowledge about the positive functioning that results from a subjective sense of well-being (SWB) in terms of happiness and life satisfaction (Diener et al., 2018), including among people exposed to traumatic events (Fredrickson et al., 2003) and daily hassles (Luhmann et al., 2013). This suggests that SWB may enable resilient outcomes in people exposed to past or ongoing adversities. However, there is a gap in the literature about the over-time predictors and outcomes of SWB among URY and other refugee youth. Hence, the overall aim of the present study is to examine the longitudinal associations between SWB and two taxing daily hassles specific to the acculturation context: perceived discrimination and ethnic identity crisis (acculturation hassles).

The present study is based on a dataset with three waves of repeated measures collected from URY, which is rare to obtain from samples of unaccompanied refugee minors and youth. Such data facilitates an examination of the over-time effects of daily hassles on SWB, as found by McCullough et al. (2000), and in the review by Proctor et al. (2009). Moreover, furthering research showing bidirectional effects of SWB and health outcomes (Diener et al., 2018) with major life events and daily hassles (Luhmann et al., 2013), we also explore the potential of SWB to lessen the amount of acculturation daily hassles experienced. Consequently, this study aims to provide new knowledge about SWB among youth in extremely vulnerable situations and supplement the literature that investigates SWB as a resilience factor in other populations (Fredrickson et al., 2003). Our study can add much needed knowledge to the growing scholarly literature on psychological adjustment among unaccompanied refugee minors and youths (El Baba and Colucci, 2018), and provide a better understanding of their psychosocial adaptation and integration into new and unfamiliar contexts. Moreover, a better understanding of healthy adaptation among URY may advance policies and practices to scaffold social contexts that promote positive developmental trajectories in the resettlement countries.

### Subjective Well-Being Among Children and Youth

SWB is a multidimensional construct, covering people’s emotional responses, as well as their cognitive evaluation of how satisfied they are with life in general or with specific life domains (Diener et al., 1999). There is a moderate degree of overlap between people’s satisfaction with important life domains such as school, relationships, and economy, and global assessments of life in general, implying that these constructs tap into similar, but slightly different processes (Huebner, 2004). Large scale population-based studies tend to employ global measurements such as the Cantril ladder (Cantril, 1965) or Diener’s Satisfaction with Life Scale (Diener et al., 1985), e.g., (Inchley et al., 2020). Research with smaller groups especially of children and youth, often involves a life domain approach (Huebner et al., 1998; Kim and Park, 1999).

Research on young people’s SWB has shown that individuals who report high levels of SWB in terms of life satisfaction, have better interpersonal relationships, exhibit less antisocial and violent behavior, engage in healthier behavior, and perform better academically than youth with lower levels of SWB (Proctor et al., 2009; Diener et al., 2018). Aggregated findings from 200,000 adolescents in Europe and Canada showed a general high level of SWB as measured by the Cantril ladder, with average country/region scores ranging from 7.2 to 8.6 on a scale from 0 to 10. Girls reported lower SWB than boys, and older adolescents had lower levels of SWB than younger study participants (Inchley et al., 2020).

The level of SWB is also generally high among ethnic minorities and immigrant background youth, but findings are inconsistent with respect to variation in level of SWB between immigrant/ethnic minority and non-immigrant/ethnic majority samples (Proctor et al., 2009). Delaruelle et al. (2021) in a study based on data from the 2017/18 Health Behaviour in School-aged Children (HBSC) study (*n* = 63.425) used the Cantril ladder to assess immigrant-status variation in the level of dissatisfaction among the students. Compared to non-immigrants 1st and 2nd generation immigrant students reported significantly higher levels of dissatisfaction, however the differences were small. Sam (1998), comparing immigrant background adolescents with origins from Chile, Vietnam, Turkey, and Pakistan with ethnic Norwegians using a 1 to 5 scale version of Diener’s Satisfaction with Life scale, did not find variation in the level of SWB between ethnic Norwegians and any of the groups. Neto (2001) employed the same version of Diener’s measure to compare immigrant background youth from Angola, Cape Verde, and India with ethnic Portuguese youth. Only Angolan background participants had lower SWB than the ethnic Portuguese. But the differences in mean levels between the three immigrant groups were not significant. Findings from the United States showed that Caucasians reported higher levels than African-Americans on a domain specific measure of life satisfaction (Huebner et al., 2004). Across these studies, the associations between demographic variables such as gender, age, and length of stay in the receiving countries were inconsistent. In summary, differences in level of SWB do not seem to be a matter of immigrant vs non-immigrant background or ethnic minority- vs ethnic majority background. Instead, variation between groups appear to run along national origin and individual ethnic groups, with additional variation between receiving countries.

The literature on SWB among refugee children and youth in general, and *unaccompanied* refugee minors and youth in particular, is scarce (Yoo et al., 2018; Bajo Marcos et al., 2021). However, studies have found significant reciprocal associations between SWB in terms of life satisfaction and major depression (Fergusson et al., 2015) and post-traumatic stress disorder, PTSD (Karatzias et al., 2013), suggesting reduced SWB among them. Hence, it is noticeable that in a cross-sectional, comparative study based on the same sample as the current study, Seglem et al. (2014) showed that the level of SWB was equal among URY, other immigrant background and ethnic Norwegian youth despite URY reporting significantly more depressive symptoms. A study among *recently arrived* asylum-seeking and refugee minors found alarmingly low levels of SWB among them, below the suggested cut-off score for low SWB (Solhaug et al., 2021). There is a need for more studies before we can make any conclusions regarding the level of SWB among unaccompanied refugee children and youth and factors that are causing over time changes.

### Daily Hassles and Subjective Well-Being in Acculturation Context

The most frequently studied predictors of SWB are socio-economic factors, self-concept, psychopathology, mental health problems and family relations, however, a few studies have also included risk factors such as major life events and daily hassles (McCullough et al., 2000; Vera et al., 2011; Orkibi and Dafner, 2016; Yoo et al., 2018). Major life events refer to dramatic experiences that are often limited in time such as the death of a close person, serious accidents or illnesses (DeLongis et al., 1982). Daily hassles on the other hand are defined as ‘irritating, frustrating, distressing demands that, to some degree, characterize everyday transactions with the environment’ (Kanner et al., 1981). When daily hassles occur frequently, are ongoing or accumulate, they increase the risk for more mental health problem and lower SWB (Kanner et al., 1981; Dumont and Provost, 1999; McCullough et al., 2000; Gaudet et al., 2005). However, a more recent line of research has accounted for more complex relations between SWB and various risk factors thereof; e.g., Luhmann et al. (2013) identified an ameliorating prospective effect of SWB on major life events whereas Fergusson et al. (2015) demonstrated over-time bidirectional effects between SWB and various mental health problems. Following up on these findings, the present study examines the reciprocal effects between SWB and daily hassles.

Acculturation is mostly used to describe the developmental processes through which ethnic minority and immigrant populations adapt to multicultural contexts and acquire the necessary competence to succeed both within their heritage and the majority culture (Berry, 1997; Nguyen and Benet-Martínez, 2013; Oppedal and Toppelberg, 2016).

In multicultural societies, people are exposed to two broad categories of daily hassles. Young people may experience g*eneral* hassles, that are independent of the cultural diversity of the developmental context, such as problems related to school achievement and conflicts in interpersonal relationships. Seglem et al. (2014) has shown that general daily hassles were negatively associated with SWB among URY, other immigrant and ethnic Norwegian youth, and that URY reported significantly more general hassles than the other youth groups. Nevertheless, the differences in level of SWB did not differ between these groups (Seglem et al., 2014).

In addition to such general hassles, people face a variety of *acculturation* hassles unique to multicultural developmental contexts, that are associated with developmental tasks that must be solved (e.g., formation of an ethnic identity), interpersonal relationships (e.g., ingroup and outgroup hassles, discrimination) or values (e.g., value-gap) (Schwartz et al., 2010; Titzmann et al., 2011; Keles et al., 2017). Research has confirmed the association of acculturation hassles with symptoms of anxiety and depression (Vinokurov et al., 2002; Titzmann et al., 2011; Keles et al., 2015). However, with the exception of perceived discrimination, there is a knowledge gap concerning the association between SWB and acculturation hassles, in particular among URY. Hence, in the present study, we include two dimensions of acculturation daily hassles of relevance to SWB, namely perceived ethnic-based discrimination and ethnic identity crisis (Keles et al., 2015).

### Subjective Well-Being and Perceived Discrimination

Ethnic-based discrimination is intrinsic to culturally diverse societies, and it is the most frequently studied acculturation risk factor of SWB among refugee and other immigrant-background youth (Sam, 1998; Neto, 2001; Verkuyten, 2008; Proctor et al., 2009; Vera et al., 2011; Benner et al., 2018; Yoo et al., 2018; Bajo Marcos et al., 2021). Despite legislation and policies to prevent its occurrence, more than half of the population in the European Union believe ethnic discrimination to be widespread in their countries (de Groot, 2021). Norway is not an exception to this, and as much as 80% of the population believe that discrimination against immigrants occur to “some” or a “great” extent (Brekke and Mohn, 2018).

The economic cost of ethnic discrimination to society is substantial in terms of loss of disability adjusted life years (DALY) due to the association of discrimination with a high burden of disease, particularly in terms of mental health problems and disorders (Elias and Paradies, 2016). Ethnic-based discrimination is associated with negative academic outcomes in terms of lower school grades, school engagement and motivation, in addition to lower subjective well-being experiences, for a review, see Benner et al. (2018). Previous studies that examined predictors of perceived ethnic-based discrimination have shown that the more symptoms of depression and anxiety youth reported, the stronger they perceived being discriminated against (Phinney et al., 1998; Neto, 2006). However, as these studies were cross-sectional, the causal direction cannot be verified. Nevertheless, high levels of anxiety and depression symptoms are expected to lead to a negative view of the world, and a greater likelihood to attribute people’s actions as discrimination (Phinney et al., 1998). In contrast, a more positive view of oneself and life in general as implied by SWB, would be related to a generally positive interpretation of real-world events, and lesser likelihood of attributing people’s behavior as discrimination (Phinney et al., 1998). Based on this, in the present study we assess the potential of SWB to reduce the level of perceived discrimination over time, in addition to examining the detrimental effect of discrimination on SWB.

### Subjective Well-Being, Ethnic Identity Development and Ethnic Identity Crisis

A major developmental task among immigrant-background youth is to resolve issues of ethnic-cultural belongingness and identification (Umaña-Taylor et al., 2014). A strong and coherent heritage culture ethnic identity is associated with SWB and a host of other positive psychosocial and academic outcomes (Smith and Silva, 2011; Rivas-Drake et al., 2014; Yoo et al., 2018). Based on the theories of Erikson (1968) and further theoretical formulations of Marcia (1980), ethnic identity development is typically described in terms of processes of exploration and commitment/resolution (Phinney, 1989; Umaña-Taylor, 2015). When these developmental processes have been examined individually in relation to SWB, exploration tends to have a negative effect on SWB, in contrast to the positive effects of commitment (Yip et al., 2019). This is probably because exploration is characterized by uncertainties regarding ethnic belongingness, and how you see yourself as part of an ethnic group (Phinney, 1992). In contrast, identity achievement and resolution represent a strong and coherent sense of one’s ethnicity and ethnic-cultural group belongingness that is associated with a coherent overall identity. Consequently, a diffuse ethnic identity resulting from very little or no exploration and commitment, was associated with the poorest, and an achieved ethnic identity following high exploration and high commitment with the most favourable psychological adjustment outcomes (Phinney, 1989).

In culturally diverse contexts, young people also explore their experiences, feelings, and thoughts about their belongingness to the majority culture (Phinney et al., 2001). Youth with strong heritage and majority culture identities have typically reported more positive psychosocial outcomes, while low scores within both ethnic identity domains were related to unfavorable outcomes (Phinney et al., 2001).

Young people who are unable to explore their ethnic identity in constructive ways, or struggle to choose and commit to heritage or majority ethnic-cultural roles and identities, may experience an ethnic identity crisis (Oppedal et al., 2004). Previous research has shown that going through an ethnic identity crisis was associated with higher levels of mental distress and lower levels of SWB (Oppedal et al., 2004, 2005; Ramberg et al., 2016). Moreover, changes in ethnic identity crisis predicted changes in depressive symptoms, suggesting co-developmental processes between these psychological phenomena (Oppedal et al., 2004). However, the longitudinal associations between an ethnic identity crisis and SWB has so far not been investigated.

Perceived discrimination is theoretically (Tajfel, 1982) and empirically correlated with ethnic identity exploration and with ethnic identity crisis (Oppedal et al., 2005; Umaña-Taylor and Updegraff, 2007). Meca et al. (2020) demonstrated that perceived discrimination also predicted ethnic identity exploration over-time, hence we will explore if a similar longitudinal effect may be present in relation to ethnic identity crisis. Additionally, we suggest a bidirectional, cross-lagged relation between perceived discrimination and ethnic identity crisis, as an ethnic identity crisis may sensitize youth to experiences of discrimination (Meca et al., 2020).

### The Present Study

In summary, building on extant research findings to expand current knowledge about SWB and acculturation among unaccompanied refugee minors and youth, the overall aim of the present study is to examine the longitudinal interrelations between SWB, perceived discrimination and ethnic identity crisis. More specifically, we examined

(1) the stability and change in SWB, discrimination and ethnic identity crisis (i.e., the auto-regressive effects)
(2) their co-development and cross-lagged relations (e.g., whether SWB predicts subsequent changes in discrimination and ethnic identity crisis, and vs.)

To exclude potential confounding between PTSD and SWB, we included intrusive symptoms, a main dimension of PTSD, resulting from exposure to war-related traumatic events as a co-variate in the analyses, in addition to gender, age, and length of stay since arrival in Norway.

## Materials and Methods

### Participants

The target group for this study was 581 unaccompanied refugees who participated in the second wave (W2) of the longitudinal, population-based project “Social support, coping and mental health among children and youth who arrived in Norway as unaccompanied minor asylum-seekers”^1^. The project was conducted by the Norwegian Institute of Public Health (NIPH).

The Norwegian Directorate of Immigration (NDI) provided contact information for the 4208 unaccompanied asylum-seeking minors ≥ 13 years of age who were granted a residence permit in Norway between 2000 and 2011. Based on available economic resources, all known unaccompanied refugees in 41 municipalities nationwide, that differed in demographic and geographic characteristics, were targeted, *n* = 1,685. Recruitment and W1 data collection started in 2006 and continued until 2011, with three subsequent follow-up data collections one, two and three years, respectively, after baseline.

Letters inviting the youth to participate were sent to their homes and if they were less than 16 years, also to their legal guardians. Of the total invited sample 476 refugees (28.25%) were not possible to locate; 47 (3.9%) actively declined participation, and 218 (18.0%) youth who confirmed their participation did not show up on the day of data collection. The study sample of 948 participants represented 78.4% of the final sampling frame. The few differences between participants and all non-participants of the population-based sample reflected the variation over time in the flow of unaccompanied minors. We excluded 30 participants with missing values on most items, yielding a final sample of *n* = 918. From this sample 581 (63.3%) also participated in second (W2), 227 (24.7%) in third (W3) and 139 (15.1%) in fourth/last (W4) data collections. As the measure of SWB was not included in the W1 questionnaire, we employ data from W2, W3, and W4 in the current study, referring to them as T1, T2, and T3, respectively in this study.

The majority (82%) of the participants were male, in line with the fact that most unaccompanied children migrating are boys. The mean age of the participants at T1 was 20.09 (*SD* = 2.61) years. Their average length of stay in Norway at the time of data collection was 4.63 years (*SD* = 2.40). Participants originated from a total of 33 different countries, mainly Afghanistan (49.6%), Somalia (10.8%), Iraq (7.1%), and Sri Lanka (7.1%).

The main reason for the substantial attrition was that the funding was unexpectedly discontinued in 2012, in addition to extensive mobility among these youth. Attrition analyses conducted based on the original W1, W2, and W3 data (Keles et al., 2017) did not show significant differences between participants and non-participants on background demographic data and study variables such as symptoms of depression and post-traumatic stress, or on indices of acculturation and general hassles.

Follow-up attrition analyses examining potential variations in sample and main study variables of the present study, supported the notion that non-participation was not selective. The distribution of age, gender, and length of stay was similar among participants in T1, T2 and T3, and non-participants. However, there were more unaccompanied minors from Afghanistan among non-participants at T3, than among the participants. Thus, in the T1-sample, 50.8% was Afghans, while in the T3-sample they represented 36.7%. This was due to the unaccompanied minor Afghans that arrived in 2009, who were recruited in 2010 and 2011, but could not be followed-up when the funding came to a halt.

We further compared the level of SWB, perceived discrimination and ethnic identity crisis at T1 and T2 among subsequent participants and non-participants, with non-significant results for all analyses. Consequently, we concluded that non-participation was not selective.

### Procedure

The Regional Committee for Medical and Health Research Ethics and the Norwegian Data Inspectorate approved the study. Participation was contingent on written consent. If participants were less than 16 years old, written consent was also obtained from their legal guardians. The approved ethics application included a security protocol with procedures if the data-collection should reveal unmet needs for psychosocial support among the participants.

The data collection was conducted in the local communities where the youths lived. At each annual follow-up wave, they gathered in small groups at a location familiar to them in each of the 41 municipalities, such as a group home or library, to fill in self-report questionnaires. Trained research assistants supported the youth during the data collection in line with the standardized protocol, which included standardized explanations of difficult concepts and English translation of the questions, in addition to procedures for the data collection. The questionnaire took about 1.5–2 h to complete. None of the participants took advantage of the offer to have translators present, who could read the questions in their mother tongue. Participants received a gift voucher, worth approximately €15 at T1 and €40 at T3, as a compensation for their time.

### Measures

The NDI provided register information about gender, national origin, birth date, arrival date, and date and place of resettlement.

#### Subjective Well-Being

Six items tapping satisfaction with different life domains found to be relevant for adolescents were used to assess SWB (Kim and Park, 1999). Respondents were asked to indicate how satisfied they were with school grades, appearance, body, social life, relationship with friends, and economic situation on a 7-point Likert-type scale from 1 (*very dissatisfied*) to 7 (*very satisfied*). The items were combined to a mean score ranging from 1 (*low*) to 7 (*high*). Cronbach’s alpha for the total scale varied between.76 and.80 across timepoints. After transforming the 1–7 score range into the often used 0–10, we used the traditional cut-off of 6 to indicate low SWB.

#### Ethnic Identity Crisis

A six-item scale was developed to measure difficulties in choosing and committing to an ethnic role among immigrant youth (Oppedal et al., 2004, 2005). The sample scale items are: “It is difficult to choose whether I should live the way young people in Norway do” and “It is difficult to decide whether I should live the way young people from my home country do.” The participants rated each item on a four-point Likert type scale, ranging from 1 (*strongly disagree*) to 4 (*strongly agree*). Cronbach’s alphas for the total scale varied between 65 and 82 across timepoints. Scores > 2 to implies high levels of ethnic identity crisis.

#### Perceived Discrimination

A five-item scale was used to measure youth’s experience of ethnic based discrimination, ranging from having been treated unfairly to having been attacked because of one’s cultural background (Berry et al., 1993). Sample items included “I have been teased or offended because of my ethnic background” and “I have been threatened or attacked because of my cultural background.” The respondents rated each item on a four-point Likert-type scale, ranging from 1 (*strongly disagree*) to 4 (*strongly agree*). Cronbach’s alphas for the total scale varied between 71 and 79 across timepoints. Scores > 2 suggests high levels of perceived discrimination.

#### Intrusive Post-traumatic Stress Symptoms

Impact of war-related trauma was assessed at T1 by three items about exposure to war first-hand and resulting health outcomes in terms of intrusive symptoms as memories and nightmares. Based on the responses of the participants, they were given a score of 0 (*no war or no symptoms*), 1 (*experienced war, 1 symptom*), or 2 (*experienced war, 2 symptoms*).

### Statistical Analyses

Conventional analyses were carried out in SPSS version 26, while Mplus 8 (Muthén and Muthén, 2017) was used to examine the longitudinal association among subjective well-being, discrimination and ethnic identity crisis. The robust maximum likelihood (MLR) estimator, which uses the FIML method (full-information maximum likelihood), was preferred to accommodate non-normal item distributions, missing data and the declining response rate with time. FIML is highly recommended with missing longitudinal data (Graham, 2009). In addition to the chi-square statistic which is sensitive to sample size (Bentler, 1990), other fit indices were consulted: the root-mean-square error of approximation (RMSEA), the comparative fit index (CFI), and the standardized root-mean-square residual (SRMR). West et al. (2012) suggest that CFI ≥ 0.95, RMSEA ≤ 0.05, and SRMR ≤ 0.06 represent a well-fitting model, while CFI ≥ 0.90, RMSEA ≤ 0.08, and SRMR ≤ 0.10 represent an adequately fitting model (West et al., 2012).

The longitudinal associations among the study variables across time were analyzed using auto-regressive (AR) cross-lagged models. Two sets of regression paths were specified in the AR analyses: auto-regressive paths indicating stability (e.g., subjective well-being T1 to subjective well-being T2), and cross-lagged paths (e.g., subjective well-being T1 to discrimination T2 and to ethnic identity crisis T2, and vice versa). First, a full reciprocal model that had cross-lagged paths going in both directions (e.g., subjective well-being T1/T2 to discrimination T2/T3, and discrimination T1/T2 to subjective well-being T2/T3) among three study variables was freely estimated. Then, a full reciprocal model in which the cross-lagged paths were constrained to be as equal, thus indicating an equal cross-lagged association across time, was estimated. As the latter model is nested within the first model, they were compared using an adjusted chi-square difference test due to the use of the MLR estimator (Satorra and Bentler, 2001). If the worsening in model fit of the nested SEM model was non-significant, it was preferred for reasons of parsimony as it explains the data comparably well.

The impact of war-related trauma as well as gender, age, and participants’ length of stay at the time of the first data point (T1) were added as covariates while estimating structural coefficients.

## Results

### Descriptive Statistics

Table 1 presents the estimated correlations, means and standard deviations among the observed study variables at each timepoint.

**TABLE 1:** Descriptives of the measures and intercorrelations among the observed variables.

	1	2	3	4	5	6	7	8	9	10	11	12	13
1. SWB T1	(–)	0.446***	0.308***	–0.185***	–0.203**	–0.376***	–0.196***	–0.096	–0.194*	–0.089*	0.047	0.137**	–0.070
2. SWB T2		(–)	0.438***	–0.241***	–0.332***	–0.360***	–0.146*	–0.179**	–0.275**	–0.038	–0.074	0.020	–0.107
3. SWB T3			(–)	–0.020	–0.323***	–0.227**	–0.199*	–0.072	–0.049	–0.091	0.057	0.114	–0.089
4. PD T1				(–)	0.279***	0.347***	0.466***	0.179**	0.240**	–0.040	0.070	0.068	0.133**
5. PD T2					(–)	0.461***	0.257***	0.404***	0.307***	–0.014	0.087	0.046	0.077
6.PD T3						(–)	0.321***	0.172	0.436***	0.065	–0.129	–0.121	0.092
7. EIC T1							(–)	0.376***	0.281***	–0.057	–0.015	–0.068	0.115**
8. EIC T2								(–)	0.296***	0.035	0.069	0.016	0.090
9. EIC T3									(–)	–0.007	–0.140	–0.192*	0.220*
10. Gender?										(–)	–0.121**	–0.030	0.011
11. Age											(–)	0.792***	0.033
12. Stay												(–)	0.029
13. IWRTE													(–)
M	5.37	5.27	5.35	1.80	1.78	1.83	2.10	2.04	2.09	–	20.09	4.63	0.77
SD	0.98	1.09	1.10	0.64	0.58	0.65	0.66	0.66	0.64	–	2.60	4.40	0.87

SWB, subjective well-being; PD, perceived ethnic-based discrimination; EIC, ethnic identity crisis; Stay, length of stay since arrival in Norway; IWRTE, impact of war-related traumatic events; T1–T3, represent data over 3 observations. Males were coded as 1 and females as 2. *p < 0.05, **p < 0.01, ***p < 0.001.

**FIGURE 1:**
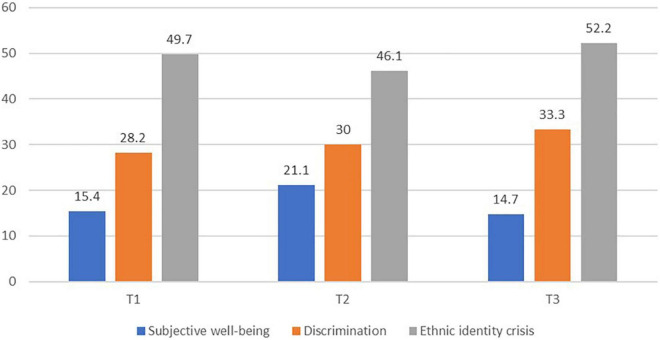
Percentages of low subjective well-being, high discrimination and high ethnic identity crisis across timepoints. Tl–T3 represent data over 3 observations.

An inspection of the mean level of the study variables across timepoints demonstrated that on average the group level of SWB, as well as discrimination and ethnic identity crisis was stable and high over time. To further examine the group level stability, we calculated the prevalence of low SWB and high discrimination and ethnic identity crisis among the participants across timepoints. The domain specific measure of life satisfaction employed to assess SWB in the present study does not have a validated cut-off score that distinguishes between low and high life satisfaction. For comparison purposes, we therefore transformed the SWB-scores, ranging from 1 to 7, into a 0 to 10 scale, such as the Cantril ladder which is often used in national well-being surveys (e.g., the World Happiness Report). The mean scores on the transformed scale were 7.28 (T1), 7.12 (T2), and 7.25 (T3). The cut-off score of ≥ that 6 is often used on this scale to distinguish high from low SWB (Levin and Currie, 2014) provided a prevalence of low SWB in our sample of 15.4, 21.1, and 14.7% on the three timepoints (Figure 1). Scores above the midpoint of 2 were used to assess the prevalence of high discrimination and ethnic identity crisis at each timepoint. As can be seen from Figure 1, the prevalence of youth being exposed to discrimination and experiencing an ethnic identity crisis is quite high and stable over the years.

The correlations between SWB and discrimination and ethnic identity crisis were negative across all timepoints, while discrimination and ethnic identity crisis were positively correlated across all timepoints.

### Auto-Regressive Cross-Lagged Model (AR)

The full reciprocal model, the free model with no constrained cross-lagged paths (Model 1), provided acceptable fit to the data [robust χ^2^_(33)_ = 55.40, *p* = 0.009; CFI = 0.939; RMSEA = 0.037, 90% CI (0.019, 0.054); SRMR = 0.070]. Constraining all twelve cross-lagged paths as equal (Model 2) (e.g., all cross-lagged paths going from SWB to discrimination and ethnic identity crisis, and vice versa as equal) did worsen the model fit significantly (ΔSB χ^2^_(11)_ = 34.05, *p* < 0.001). Relaxing the constraints by requiring the cross-lagged paths from one variable to the other constrained to be equal over time (Model 3) (e.g., constraining the two cross-lagged paths going from SWB to discrimination as equal, and the two cross-lagged paths from discrimination to SWB as equal), did not worsen the model fit ΔSB χ^2^_(6)_ = 10.71, *p* = 0.098). Hence, the cross-lagged coefficients were equal over time, but of different size depending on the direction from SWB, discrimination, and ethnic identity crisis. We also ran a model with constrained correlated residuals over time (Model 4), indicating an equal co-development between each pair of variables across time. Constraining residual correlations equal did not worsen the model fit ΔSB χ^2^_(12)_ = 19.99, *p* = 0.067). The fit indices for each model are presented in Table 2.

**TABLE 2:** Fit indices of the auto-regressive cross-lagged models.

	Robust χ^2^	df	RMSEA	CFI	SRMR	Model comparison	Δχ^2^	Δdf
M1	55.396	33	0.037	0.939	0.070			
M2	88.955	44	0.045	0.877	0.099	M1 vs. M2	34.05***	11
M3	66.048	39	0.037	0.926	0.076	M1 vs. M3	10.71	6
M4	75.386	45	0.037	0.917	0.083	M1 vs. M4	19.99	12

M1, Reciprocal Model (free model); M2, Reciprocal Model (all cross-lagged paths constrained equal); M3, Reciprocal Model (cross-lagged paths from one variable to the other constrained equal over time); M4, Reciprocal Model (cross-lagged paths from one variable to the other constrained equal over time and residual correlations between variables constrained equal over time). Δχ^2^ = Satorra-Bentler corrected chi-square difference test for nested models. ***p < 0.001.

**FIGURE 2:**
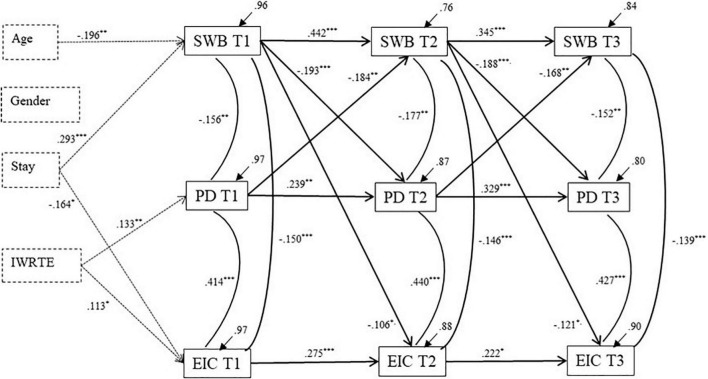
Final model (Model 4). IWRTE, Impact of war-related trauma; Stay, Length of stay. SWB T l-SWB T3, PD T l -PD T3, and E CT l-EICT3 represent data over 3 observations for subjective well-being, perceived ethnic-based discrimination and ethnic identity crisis, respectively. The residual variance components (error variances) indicate the amount of unexplained variance. Standardized parameter estimates were reported only for the significant paths. *p < 0.05, ^**^p < 0.01, ^***^p < 0.001.

Figure 2 displays the final cross-lagged model with only significant paths (Model 4). The stability (auto-regressive) coefficients for both SWB, discrimination and ethnic identity crisis were of a low-to-moderate size. This implies that despite the over-time stable group mean levels of these variables and almost similar percentage reporting low SWB and high perceived discrimination and ethnic identity crisis, there was substantial within person variation in the reported levels of these factors across the timepoints.

### Cross-Lagged Effects and Co-development

The significant paths from SWB to discrimination as well as ethnic identity crisis from T1 to T2, and from T2 to T3, were negative, hence indicating that higher levels of SWB at one point in time predicted a significant decrease in perceived discrimination as well as in ethnic identity crisis at the subsequent time.

The cross-lagged paths from perceived discrimination to SWB were also significant and negative. That is, higher levels of discrimination at one point in time predicted a significant decrease in SWB at the subsequent time. Hence, there was a reciprocal association between SWB and discrimination over time. In contrast, ethnic identity crisis did not predict subsequent changes neither in SWB nor in perceived discrimination over time, indicating that there were no cross-lagged effect involving ethnic identity crisis.

In addition to the cross-lagged paths, we observed substantial co-development across time, as indicated by the correlated residuals. That is, after controlling for effects from previous time points (i.e., stability and cross-lagged-effects) there were significant correlations between the changes in the included variables at T2 and T3. Thus, when SWB increased over time there was also a parallel decrease in perceived discrimination and identity crisis. The strongest co-development was observed between ethnic identity crisis and perceived discrimination, with correlations of.44 and.43, at T2 and T3, respectively.

### The Co-variates

There was a significant negative effect of age on the initial level of SWB, implying that younger participants had higher initial levels of subjective well-being (β = –0.196, *p* = 0.006). Length of stay, on the other hand, was associated both with initial levels of SWB and of ethnic identity crisis, but in opposite directions (β = 0.293, *p* < .001, and β = –0.164, *p* = 0.030, respectively). That is, higher initial levels of SWB were related to a longer stay in Norway, however, lower initial levels of ethnic identity crisis were related to a longer stay in Norway. Impact of pre-migration war-related trauma was positively related both to initial levels of perceived discrimination and to ethnic identity crisis (β = 0.133, *p* = 0.004, and β = 0.113, *p* = 0.015, respectively), but not to SWB. Gender was not a significant covariate in the model.

## Discussion

The current study is the first to examine the bidirectional over-time relations (autoregressive, cross-lagged, and reciprocal), in addition to the co-developmental associations between SWB and acculturation daily hassles (i.e., discrimination and ethnic identity crisis) in a multi-ethnic sample of URY. The rationale for examining two conspicuous acculturation hassles in relation to SWB, was to get knowledge about the potential of SWB to promote resilient outcomes among youth in highly vulnerable situations. The findings demonstrate complex over-time relations between the three processes and the potential of SWB to protect against experiences of discrimination and challenging ethnic identity development.

The analyses yielded non-significant effects from suffering from intrusive symptoms following exposure to war-related traumatic events (IWRTE) on initial levels of SWB. This is unexpected as findings from both community samples and studies involving refugees have demonstrated significant associations between SWB and post-traumatic stress-symptoms (PTSS) (Karatzias et al., 2013; Choi et al., 2017). Also, previous research findings from the same sample, showed significant effects of IWRTE on initial levels of depressive symptoms, but one year earlier from this current study, at the first data collection (W1) (Keles et al., 2017). It may be that as time passes the association between IWRTE and SWB is indirect, through e.g., length of stay in the country of refuge. Future studies should examine the over-time relations of SWB with IWRTE and other measures of PTSS among refugee youth at various timepoints and transitions in the resettlement process. This may provide valuable knowledge about the susceptibility of SWB to mental trauma reactions in different stages of the refugee process from the claim for asylum is lodged, until the young people are well-established in their new countries of residence.

### Level of Subjective Well-Being

The mean level of SWB was high among the URY. As different measures, partly with different response formats, have been used to capture different aspects of SWB, scores are not necessarily comparable across studies. Our strategy of transforming scores into a 0–10 scale enables comparisons between scales of high content overlap. This approach contributes to putting our results in a context of previous findings, but the findings as such pertain to the constructs in focus. On the transformed scale with scores ranging from 0 to 10, the group mean varies between 7.12 and 7.28 at the three timepoints, which is well above the often-used cut-off score ≥6 of the Cantril ladder indicating high SWB (Levin and Currie, 2014). The scores are also close to the Norwegian population mean of 7.55 (Helliwell et al., 2019), and within the range of average scores among representative samples of adolescents from 44 European countries plus Canada, that varies between 7.2 and 8.6 (Inchley et al., 2020). A national population based Norwegian survey among lower and upper secondary school students (grades 8–13) yielded a prevalence of “dissatisfied” youth with SWB scores < 6 of 15% on the Cantril ladder (Bakken, 2020), also very similar to the prevalence rates of the presents study.

The high level of SWB among these URY is striking, bearing in mind the demonstrated significant reciprocal associations between SWB and mental health problems (Fergusson et al., 2015), and the high prevalence of depressive symptoms among these youth (Keles et al., 2018). However, findings from a recent study that examined the underlying gene-environment architecture of wellbeing and mental health problems, supported the notion that the two constructs are separate, and only partially overlapping (Hofgaard et al., 2021).

It should also be considered that the study participants have received protection as refugees and residence in the country. Moreover, their mean length of stay at the time of the T1 data collection was 4.63 years (Table 1). They have had the chance to regain stability in their lives by participating in school or work, and many have reestablished connections with family abroad and reconstructed social networks in Norway (Oppedal and Idsoe, 2015; Oppedal et al., 2017). Unaccompanied minors who obtain protection and residence in Norway, are followed up and supported by local refugee or child welfare services, and if needed, they are entitled to supportive measures until the age of 24 (The Child Welfare Act, 2021). Moreover, health care and education, even at tertiary level, are free in Norway, providing refugee youth with security, opportunities, and hope for the future that may contribute to their sense of well-being.

Comparison to some standard of reference, e.g., other people’s situation or one’s own aspirations, is key to how individuals assess their SWB (Cummins, 2003; Voicu and Vasile, 2014). Thus, an additional explanation to the high levels reported by the URY may be that they compare their present situation to their situation before leaving their homeland, or relative to family and friends that are left behind. A study that examined SWB among immigrants to test whether SWB reflect stable personality traits or is malleable and driven by one’s life circumstances, showed that immigrants’ level of SWB over time adjust to the population level in their destination countries in accordance with their changing living conditions (Helliwell et al., 2016). In 2019, Afghanistan, Somalia, Iraq, and Sri Lanka, ranked between 112 and 154 on the World Happiness Index, with scores ranging from 3.20 to 4.67 (Helliwell et al., 2019), well below the adjusted means of the participants in the present study, who mainly originated from those countries. Comparative studies of the level of SWB among URY across various destination countries can shed more light on the contextual factors that mould their satisfaction with life.

The data for the present study were collected well in advance of the high numbers of new arrivals of asylum-seekers and refugees to Europe in 2015, which resulted in considerable restrictions in immigration and integration policies. In contrast to the findings in the present study, a more recent study of SWB among unaccompanied asylum-seeking and refugee minors who arrived at that time, showed extremely low levels of SWB; even youth who had received a residence permit reported levels below the cut-off for dissatisfaction (Solhaug et al., 2021). The study concluded that factors both at the macro level (e.g., immigration policies) and at the micro level (e.g., daily hassles) accounted for the low levels of life satisfaction.

These contradictory findings call for more research efforts, particularly longitudinal studies of the SWB of unaccompanied as well as accompanied asylum-seeking and refugee children and youth. Distinguishing groups based on age at arrival in the destination country and length of stay, could provide valuable new insights into the over-time changes in SWB of refugee children and youth.

### Subjective Well-Being and Acculturation Hassles Over Time

The (group) means of SWB, perceived discrimination and ethnic identity crisis were high and quite stable across the three datapoints (Table 1). Notwithstanding, the autoregressive paths of the final cross-lagged model (Figure 2) were in the low to moderate range for the three processes, indicating substantial individual changes in scores over the three timepoints. However, these findings do not inform about the size and the direction of the individual score changes. Other studies have observed declining stability in SWB over one year, e.g., among high-school students in Canada (Huebner, 2004), Mexican origin youth in the United States (Willroth et al., 2021), and expecting mothers in Norway (Dyrdal et al., 2011), concluding that the stability in SWB declines over time.

The over-time negative effect of perceived discrimination on SWB is in accordance with numerous research findings documenting the detrimental effects of discrimination on multiple health, well-being, and functioning outcomes, e.g., (Carter et al., 2017; Benner et al., 2018). Nevertheless, a growing scholarly literature has also focused on the protective effects of SWB in relation to mental health problems (Fergusson et al., 2015), major negative life events (Proctor et al., 2009), and daily hassles (McCullough et al., 2000). The finding that SWB also protects against experiences of discrimination and ethnic identity crisis in URY is novel and adds to this literature. SWB is positively associated with self-esteem (Proctor et al., 2009), and Phinney et al. (1998) argues that a positive outlook on oneself and life in general is related to a generally positive interpretation of real-world events, and lesser likelihood of attributing people’s behavior as discrimination. Following this line of thought, we may expect that the longitudinal negative effect of SWB on perceived ethnic discrimination is mediated by higher levels of self-esteem.

On the other hand, research has also demonstrated that individuals who are satisfied with their lives, create and engage in positive relationships and transactions with the environment (Moore et al., 2018), and thereby may reduce the amount of ethnic-based discrimination they are exposed to and perceive. From this perspective, social supportive relationships would be a potential mediator between higher levels of SWB and over time reduced perception of discrimination.

As shown in Figure 1, struggling with one’s bicultural identity exploration and not being able to make choices about one’s ethnicity and belongingness, is common among URY, with prevalence rates of high levels around 50% at the three timepoints. However, we did not identify reciprocal cross-lagged effects between SWB and ethnic identity crisis, as was the case with perceived discrimination. Nevertheless, a similar over-time ameliorating effect of SWB as we observed with discrimination, was also present with respect to ethnic identity crisis. Research findings is consistent in demonstrating the positive associations between stronger ethnic identity, in terms of feelings of connectedness to one’s heritage culture, and indices of well-being among immigrant youth and youth with ethnic minority background (Smith and Silva, 2011). There are less studies examining the SWB correlates of identifying with the majority culture, or of bicultural identification, but it is generally accepted that affiliation with both cultures promote the most adaptive psychological outcomes (Berry, 1997; Phinney et al., 2001; Schwartz et al., 2010). There are few studies of outcomes and predictors of the ethnic (bicultural) identity of unaccompanied and other refugee youth, particularly longitudinal studies. Building on extant research findings regarding heritage and bicultural ethnic identity and the findings from the present study, one target for future research should be to explore the potential of these identity constructs to mediate the association between SWB and ethnic identity crisis.

Another important finding is the additional significant co-development between all three processes, beyond the cross-lagged effects, implying that increases in SWB is associated with decreases in both perceived discrimination and ethnic identity crisis, and vice versa. The demonstrated combination of the cross-lagged effects and the co-developmental associations highlights the potential of SWB as a resilience factor among URY facing acculturation hassles.

### Limitations

Research has shown that a high level of attrition across timepoint might bias estimates of study variable means but not of associations between variables (Gustavson et al., 2012). To examine if attrition in the present study was non-selective, we relied on analyses from a previous study that confirmed that non-participants at follow-up waves did not differ from participants at previous waves on demographic background information, level of depression or daily hassles (Keles et al., 2017). Furthermore, we conducted additional attrition analyses for the present study, which showed similar distribution of all sample characteristics and study variables, except national origin, among participants in T1, T2 and T3, and non-participants.

Due to the changing nationalities in different waves of unaccompanied asylum-seeking minors across time, there were fewer Afghan URY in the T3-sample. Participants’ ethnic-cultural background may be associated with level of SWB, discrimination and ethnic identity crisis, and with the associations among these variables. Unfortunately, our sample was too small to examine variation between ethnic-cultural groups. Such information would enhance our knowledge of how refugee youth with different ethnic-cultural background perceive their SWB, and if this is related to perceived discrimination and ethnic identity crisis similarly across groups. More research is needed to conclude about the generalizability of the present study findings beyond URY with relatively lengthy residence in Norway.

According to theory and empirical evidence, the accumulation of several daily hassles over time may haphazard young people’s health and well-being (Kanner et al., 1981; DeLongis et al., 1982; Evans and Cassells, 2014; Keles et al., 2015). Still, for this study we decided on employing two individual acculturation hassles, rather than the composite measure used in previous studies (e.g., Keles et al., 2015). In the literature, both ethnic-based discrimination and ethnic identity have been investigated as correlates of SWB among youth with immigrant background, but not longitudinally in samples of refugees (Smith and Silva, 2011; Benner et al., 2018; Bajo Marcos et al., 2021). Hence, our study contributes new knowledge about the interrelations of perceived discrimination and ethnic identity in relation to SWB, by demonstrating differentiated effects of the two acculturation hassles.

We employed a domain specific measure of SWB, that provides information about the cognitive evaluation of major life domains relevant to youth (school, self, social life, friends, and economic situation), rather than an assessment of the global, overall quality of their lives. Measures based on a domain model, require that they include all the crucial domains that contribute to overall life satisfaction (Huebner, 2004). To our knowledge, such measures have not been validated among refugee youth. Validation studies in other groups have shown correlations with global SWB measures in the moderate range, in addition to equivalence of the factor structures across various (non-immigrant) ethnic cultural groups (Huebner, 2004). However, to our knowledge, it has not been considered if the transnational realities and multicultural experiences of refugee and other immigrant youth are of importance to their life satisfaction. e.g., a differentiation between satisfaction with relations to friends and family abroad as well as in the resettlement country could add strength to the assessment of SWB and increase our knowledge of central domains of SWB in immigrant and refugee populations. There is a need for research, both qualitative and quantitative, to get knowledge about the suitability of existing well-being measures to reflect the transnational and multicultural experiences and realities of refugee and other immigrant youth.

## Conclusion

The study findings add much needed knowledge to the growing scholarly literature on psychological adjustment among unaccompanied refugee minors and youths, by providing new information about SWB and acculturation hassles among URY. Given the population-based sample and the longitudinal data from three annual timepoints, the study represents an important step forward to better understand the positive psychological adjustment and social mobility among these youth.

The study provides new knowledge about over-time stable and high levels of SWB despite correspondingly high and stable levels of perceived discrimination and ethnic identity crisis among URY at high risk for mental health problems. In combination, the identified cross-lagged effects of SWB and the co-development of changes in SWB and acculturation hassles underscore the potential of SWB as a resilience factor among URY.

One major implication of the study is the need for future studies of unaccompanied refugee children and youth to not only focus on psychopathology, but also on indices of SWB. One of the aims should be to detect SWB precursors and outcomes in addition to the mechanisms that link them together. This would provide salient knowledge to inform political policies and social worker and clinicians’ professional practices that aims at promoting URY’s individual adaptive resources.

Another major implication is that interventions to promote the psychological adaptation of URY should target their SWB and consider their multicultural developmental contexts. Rather than participation in education or work force, the goal of integration policies and practices for (unaccompanied) refugee children and youth, should be a strong sense of satisfaction with life, similar as for all citizens.

## Data Availability Statement

The datasets presented in this article are not readily available because the participants in the study have not consented to making the data available for the general public. Requests to access the datasets should be directed to the corresponding author.

## Ethics Statement

The study was reviewed and approved by the Regional Committee for Medical and Health Research Ethics. Written informed consent to participate in this study was provided by all participants. If participants were less than 16 years old, written consent was also obtained from their legal guardians.

## Author Contributions

As the PI, BO provided the data for the present study, has been responsible for all stages of the data collection, suggested topic and study questions for the manuscript that were discussed with the co-authors, and drafted the abstract, introduction, method and discussion parts, and reviewed the result section. SK conducted the analyses, wrote the result section, and reviewed and commented on all drafts of the manuscript. ER supervised on the analyses and reviewed and commented on all drafts of the manuscript. All authors contributed to the article and approved the submitted version.

## Conflict of Interest

The authors declare that the research was conducted in the absence of any commercial or financial relationships that could be construed as a potential conflict of interest.

## Publisher’s Note

All claims expressed in this article are solely those of the authors and do not necessarily represent those of their affiliated organizations, or those of the publisher, the editors and the reviewers. Any product that may be evaluated in this article, or claim that may be made by its manufacturer, is not guaranteed or endorsed by the publisher.

## Footnotes

1
www.fhi.no/studier/ungkul
